# A Dietetic Case

**Published:** 1888-07

**Authors:** A. McNeil

**Affiliations:** San Francisco


### A DIETETIC CASE.

A. McNeil, M. D., San Francisco.

(Translated, with comments, from the *Zeitschrift für Homœopathie.*)

I have repeatedly had the opportunity of corroborating the views of Hahnemann as expressed in sections 262 and 263 of the *Organon*, which says that in acute diseases (why limited to acute? McN.) the longing for certain dishes should be gratified in moderation. Hahnemann designated such longing as the "internal voice of Nature," as a sign of the "awakened impulse to sustain life." I think a report of such a case is of general interest.

A child of four years sickened with symptoms of fever of a disease of the digestive organs, to which no name could be given. During a period of four weeks, one after another, three different physicians were called, but none of them after a careful examination could make a diagnosis. The temperature arose in the evening to 40° Celsius (104 F.), and in the mornings rose to 38.5 or 39 (101.3 or 102.1 F.). The child was free from pain and failed constantly, for it was only with difficulty that he could be induced to take a little milk or a spoonful of soup. The tongue was at first moderately coated, later it was brownish, the lips were covered with brown crusts, stool suppressed, the bowels only a little distended and never painful. Typhoid it was not, for the spleen was normal. The most one could do was to speak of the case as a kind of gastric fever, for the sensorium was unaffected. All possible remedies were tried; the case, however, remained the same, and as the pulse continued to get weaker and was only for a short time improved by wine, so that death was expected every day. I sat by the bedside one afternoon and mentioned over to him every imaginable kind of food, but he shook his head at the name of everything. But when I mentioned grapes his face literally became transformed, and he raised himself painfully, looked about the room, and eagerly asked, "Where?" I promised to get them, and after running around the city I finally obtained some Spanish grapes; it was then February, and I paid two marks (about forty-five cents) for a pound. When I brought them to him he reached for them greedily. The first berries, which were quite hard, he did not chew, but swallowed them whole, so that I crushed them one by one and gave them to him. After he had eaten half a pound I stopped. That evening he slept soundly, and continued to do so till morning. Then the temperature was 38 C. (100.4 F.). He asked for more grapes, and through the day still more. The dark-yellow urine had been extraordinarily rich in the urates, looking like muddy water. In the evening the temperature rose to 38.5 C. (101.3 F.); next morning it was normal. In the next three weeks the child recovered perfectly, and became strong and well.

